# Efficiently Simulating an Endograft Deployment: A Methodology for Detailed CFD Analyses

**DOI:** 10.1007/s10439-020-02519-8

**Published:** 2020-05-11

**Authors:** Faidon Kyriakou, Craig Maclean, William Dempster, David Nash

**Affiliations:** 1 Department of Mechanical and Aerospace Engineering, University of Strathclyde, 75 Montrose Street, Glasgow, G1 1XJ UK; 2 Terumo Aortic, Inchinnan, Glasgow, PA4 9RR UK

**Keywords:** Stent-graft, Finite element analysis, Hemodynamic, Fabric, Aneurysm, EVAR, Anaconda

## Abstract

Numerical models of endografts for the simulation of endovascular aneurysm repair are increasingly important in the improvement of device designs and patient outcomes. Nevertheless, current finite element analysis (FEA) models of complete endograft devices come at a high computational cost, requiring days of runtime, therefore restricting their applicability. In the current study, an efficient FEA model of the Anaconda™ endograft (Terumo Aortic, UK) was developed, able to yield results in just over 4 h, an order of magnitude less than similar models found in the literature. The model was used to replicate a physical device that was deployed in a 3D printed aorta and comparison of the two shapes illustrated a less than 5 mm placement error of the model in the regions of interest, consistent with other more computationally intensive models in the literature. Furthermore, the final goal of the study was to utilize the deployed fabric model in a hemodynamic analysis that would incorporate realistic fabric folds, a feature that is almost always omitted in similar simulations. By successfully exporting the deployed graft geometry into a flow analysis, it was illustrated that the inclusion of fabric wrinkles enabled clinically significant flow patterns such as flow stagnation and recirculation to be detected, paving the way for this modelling methodology to be used in future for stent design optimisation.

**Electronic supplementary material:**

The online version of this article (10.1007/s10439-020-02519-8) contains supplementary material, which is available to authorized users.

## Introduction

The numerical modelling of endovascular aneurysm repair (EVAR), the minimally invasive technique of treating an abdominal aortic aneurysm (AAA), has seen significant progress over the last few years. Preliminary studies of the endograft device deployment involved both an idealized endograft and an idealized AAA.^16^,^27^ Although useful insights are achievable through this approach, such analyses can only reveal gross trends and features of the surgical technique since they fail to include key mechanical and geometrical characteristics such as stent design or graft fabric. Perhaps the first study on virtual deployment of a bifurcated endograft in a non-idealized AAA was published in 2012,^8^ where the numerical model developed was compared against an experimental endograft deployment inside a silicone aneurysm. The study is significant because it showed that a finite element analysis (FEA) can predict the global position of a post-deployment endograft. Nevertheless, the simulated device was under-expanded during deployment and at the bifurcation of the aorta produced significant errors when compared to the experimental images. Unfortunately, neither the distance (i.e., error) between the deployed struts and the predicted ones nor the runtime of the analysis was reported.

In 2015, Perrin *et al*.^24^ presented an integrated approach to delivering and deploying an EVAR endograft inside a patient specific geometry, reporting anywhere from 55 to 100 h of runtime. The technique was further developed and the following year was used to simulate the Anaconda™ endograft (Terumo Aortic, Glasgow, UK).^25^

To date, the paper of Perrin *et al*.^25^ represents the state of the art for full Anaconda™ simulation available in the literature. The full device model was validated against one *in vitro* and two *in vivo* deployments and the comparative results were deemed satisfactory, with the maximum error between the virtual and the experimental stents being generally below 5 mm. This threshold was claimed to be a commonly accepted limit practitioners use when incorporating simulations in their clinical workflow^25^ and has been used herein as well, as a benchmark. The major draw-back of Perrin *et al*. study was its computational cost, since the reported runtime was over 40 h (on a 12-core computer). Given the multiple exploratory studies necessary per patient and the number of patients treated for AAA each year (2882 were reported to undergo EVAR in 2015 in the UK alone^38^), this timeframe is very challenging for clinical practice while it seriously limits its applicability, even in product development applications.

Alongside structural analysis models, computational fluid dynamics (CFD) studies of endografts have also been developed. In 2014, the first patient-specific hemodynamic analysis of the fenestrated Anaconda™ was conducted in order to examine the drag forces acting on the device,^12^ yet the modelled endograft was idealized. As in most studies, the fabric of the device (which serves as a boundary to the blood flow) was considered to be smooth. Nevertheless, the wrinkles and folds on the surface of the graft should be expected to have an effect on the flow, altering the shear stresses or inducing micro recirculation, effects that have been connected to the formation of thrombus.^34^

Aortic endografts have higher rates of occlusion than open surgical alternatives,^21^,^31^ possibly because of the occurrence of folds within an oversized stent-graft. An occlusion within the limb of an aortic endograft is clinically significant as it can result in symptoms such as claudication, weakness and ischemia in the lower extremities. If conservative treatment using antiplatelet drugs proves ineffective, the occluded graft may require secondary intervention in the form of a thrombectomy, a bypass or further stent-graft insertion. Nevertheless, limitations in the imaging of standard follow-up procedures (like contrast enhanced CT scans) cannot allow the study of fabric folds, because of the high resolution required to capture such effects. Therefore, a stent model that could adequately capture the effect of fabric folds on the flow, would be a valuable tool in device design, and in the prediction of long-term device performance post-implant.

In this study, an efficient FEA model of the Anaconda™ endograft was developed, able to predict the deployed shape of the device in a few hours timeframe. Most importantly, the output of the structural simulation was used for the simulation of a hemodynamic analysis with a non-idealized boundary, in order for the effect of the fabric wrinkles to be examined. To the best of the authors’ knowledge, this is the first CFD study of EVAR, where fabric wrinkles obtained from FEA have been taken into account.

## Materials and Methods

**Figure 1:**
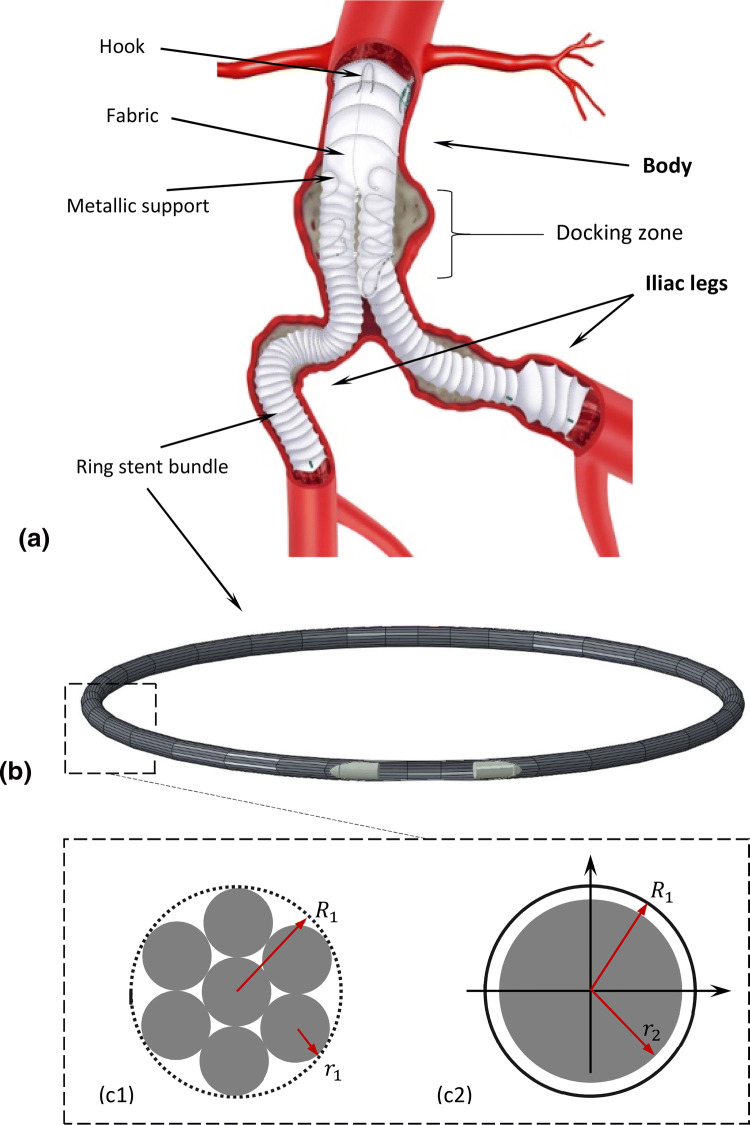
The Anaconda™ endograft (Terumo Aortic), deployed into an AAA^36^ (a). All the major building blocks of the device are highlighted. The ring stent bundle, in particular, is illustrated as modelled, with the beam and surface elements being visible (b). Regarding its cross-section, the n-wire configuration (c1) is simulated as an equivalent wire model (c2).

The Anaconda™ device consists of 3 separate modules, the body and two iliac legs (Fig. 1a), each one consisting of Nitinol rings sutured to a fabric conduit and delivered to the AAA *via* separate catheters. During EVAR, the modules are connected at the docking zone and stay fixed relative to each other. Nevertheless, in order to reduce the computational cost of simulating the interaction between those pieces, and since the focus of this study is to recreate the fabric geometry, the device developed herein was modelled as a single part. The relative position of the three modules though, was considered at the model building stage of the device, prior to the initiation of the analysis.

The analysis presented was carried out using Abaqus/Explicit (version 6.13-2, Dassault Systemes Simulia Corp., RI, USA) and all parts of the model are described below.

### Ring Model

A computationally efficient structural model of the ring stent bundles was achieved by representing the geometry and stiffness characteristics separately, similarly with a work developed earlier within our group.^15^ More specifically, circular ring bundles were constructed using beam elements (B32, 3-node quadratic beam), enclosed by surface elements (SFM3D4R, 4-node quadrilateral surface element, reduced integration) as shown in Fig. 1b. Surface elements captured the geometry of the bundle, while beam elements approximated its structural stiffness. An equivalent radius $r_{2}$ was assigned to each ring by setting it to be marginally below its respective bundle radius $R_{1}$ ($r_{2}$ = 0.96·$R_{1}$). An equivalent elastic modulus $E_{2}$ was also assigned, by equating the bending stiffness product *EI* of $n$ overlapping wires to that of 1 wire:

1 $$E_{1} I_{\text{overlapping}} = E_{2} I_{2} \Rightarrow E_{2} = E_{1} \frac{{nr_{1}^{4} }}{{r_{2}^{4} }},$$

where $E_{1}$ is the elastic modulus of the linear austenitic region of Nitinol (considered as 59 GPa^3^) and $r_{1}$ being the original radius of the wire that forms the bundle. As a result, the material used was linear elastic with ring-dependent stiffness $E_{2}$, Poisson’s ratio $v$ = 0.33 and density 6.45 g/cm^3^. The linearity of the material is an assumption made to simplify Nitinol’s superelastic behaviour and reduce the computational cost of the simulation. As the output of interest for the structural analysis is the final shape of the stent, this decision is not expected to yield significant errors. The beam and surface elements had a minimum length of twice the radius of the respective wire of each ring, according to mesh convergence results.

Apart from the bundle rings, the wire sections of the Anaconda™ include two S-shaped supports and four hooks. Both these elements though were excluded from the analysis because of their design complexity and the mild significance in the final global shape of the device.

### Fabric Model

**Figure 2:**
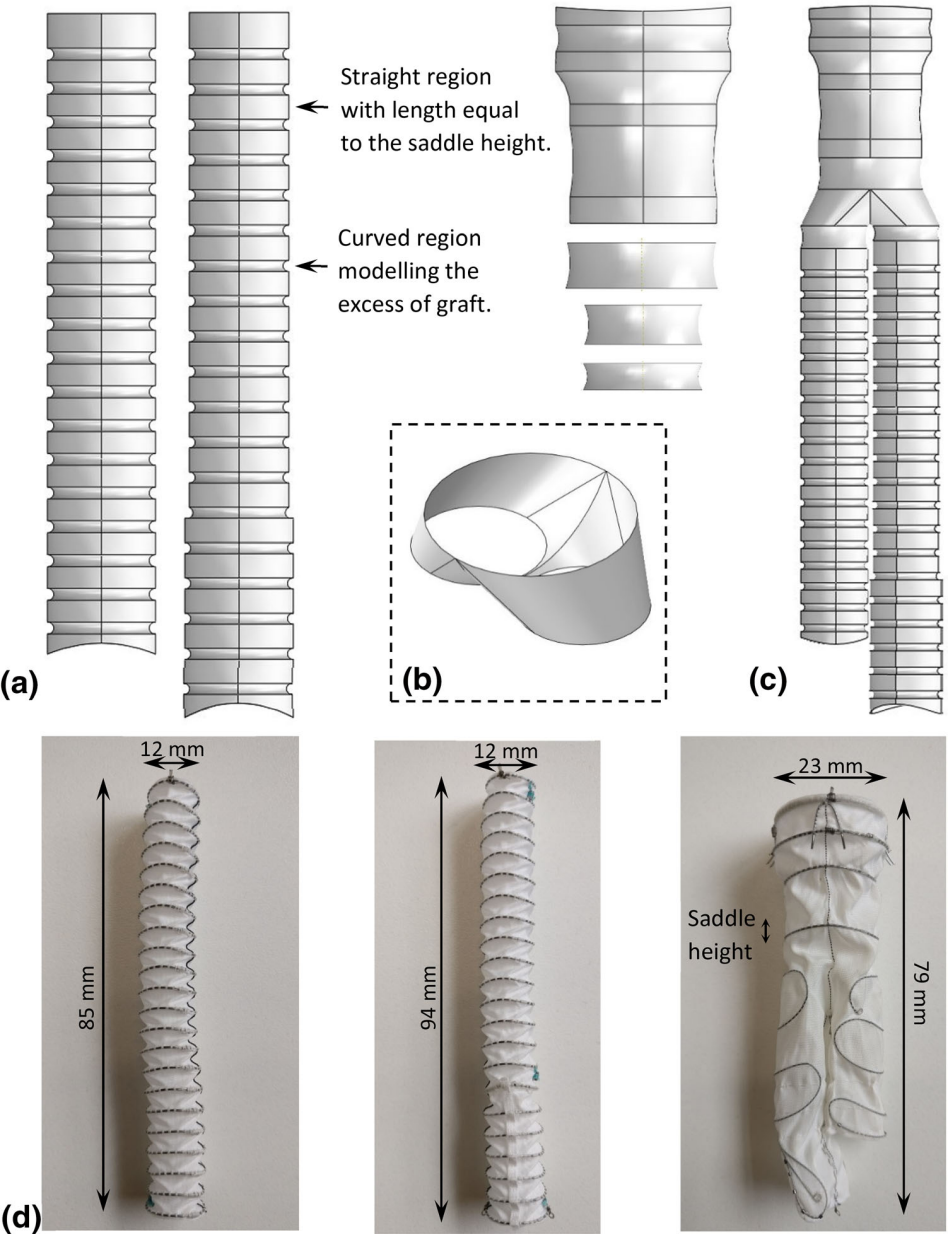
The tubular parts of the fabric were created using Abaqus (a). The distal end of the body module was imported from SolidWorks (b) and then connected to the remaining parts to form the entire graft (c). Two iliac legs and a body of a real device are also shown for comparison (d).

The ring bundles of the Anaconda™ are hand-sewn onto a bifurcated (body) or tubular (leg) fabric frame, which adopts a wrinkled shape when unpressurised, characterized by folds that are difficult to reproduce numerically (Fig. 2d). Hence, the approach followed herein to model the graft was idealized and consisted of two phases.

The first phase involved the main tubular sections of the body and leg modules (Fig. 2a). These parts have straight regions, with lengths equal to the saddle height of each ring, and curved regions, allocated to the spaces between the rings; the curves allow the excess of graft present in the resting state of the Anaconda™ to be modelled (Fig. 2d). In the second phase, the distal region of the body module was modelled. This section splits the original lumen into 2 lumens and it was designed in SolidWorks 2017 (Dassault Systèmes SolidWorks Corp) (Fig. 2b), from where it was imported into Abaqus and connected with the other fabric parts to create the final graft (Fig. 2c). The fabric was modelled using Kirchhoff thin shell elements (S4R, reduced integration scheme with enhanced hourglass control and finite membrane strain formulation) with 0.25 mm length size while the material characteristics used were taken from the literature^13^,^27^ (Table 1).

**Table 1:** Parameters of the fabric model.^13^,^27^

Elastic modulus [MPa]	55.2
Poisson’s ratio	0.46
Thickness [mm]	0.14

### Fabric Testing

For the calculation of the excess of fabric present between the rings, which corresponds to the size of the curves existing in the graft (Fig. 2a), measurements were conducted in the OLB23 Anaconda™. More specifically, the distance between the rings R1-R2 and R3-R4 was measured at the saddle points (peaks and valleys), both at rest and at full extension (Fig. S1 of Electronic Supplementary Material). These distances lay mainly in the axial direction of the endograft and were assumed to be a good approximation of the average % extension of the fabric for all the endograft modules.

**Figure 3:**
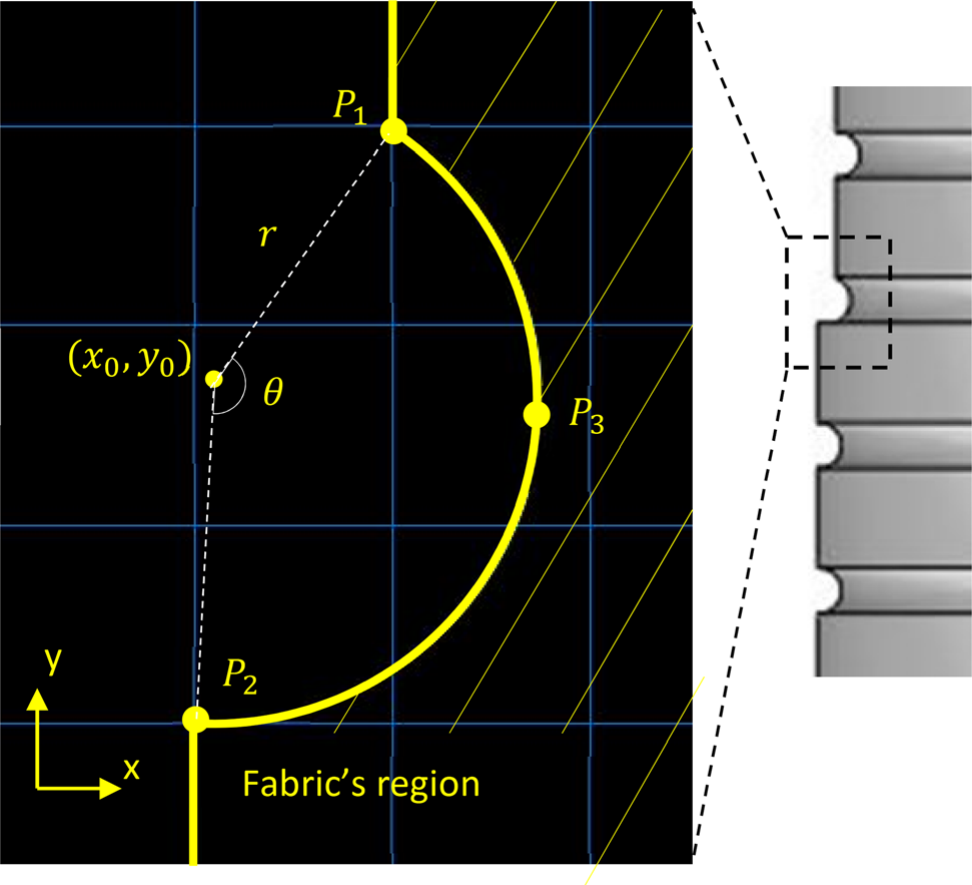
Detail of the fabric definition. The linear sections of the graft are connected by curves.

By comparing the experimentally acquired values from the fully extended state and the rest state of the graft, the available fabric slack, $L_{\text{s}}$, was calculated and, in turn, connected to the curves of the fabric model (Fig. 2a). Each one of these curves was designed as a circular section defined by 3 points (Fig. 3). And since the points at the straight region of the fabric were fixed, only 1 point controlled the excess amount of fabric between the rings ($P_{3}$ at Fig. 3).

In more detail, assume the 3 points $P_{1} \left( {x_{1} ,y_{1} } \right)$, $P_{2} \left( {x_{2} ,y_{2} } \right)$ and $P_{3} \left( {x_{3} ,y_{3} } \right)$ with $P_{1} ,P_{2 }$ given and:

2 $$y_{3} = \frac{{y_{1} + y_{2} }}{2}.$$

Then, the arc length $L$ of the circular section is controlled by $x_{3}$ alone.

If we solve the standard equation of the circle:

3 $$(x - x_{0} )^{2} + (y - y_{0} )^{2} = r^{2} ,$$

for the points $P_{1} ,P_{2} ,P_{3}$, we get for the centre $\left( {x_{0} ,y_{0} } \right)$ and the radius $r$:

4 $$x_{0} = \frac{{\left( {x_{1}^{2} + y_{1}^{2} } \right)\left( {y_{2} - y_{3} } \right) + \left( {x_{2}^{2} + y_{2}^{2} } \right)\left( {y_{3} - y_{1} } \right) + \left( {x_{3}^{2} + y_{3}^{2} } \right)\left( {y_{1} - y_{2} } \right)}}{{2\left( {x_{1} \left( {y_{2} - y_{3} } \right) - y_{1} \left( {x_{2} - x_{3} } \right) + x_{2} y_{3} - x_{3} y_{2} } \right)}},$$

5 $$y_{0} = \frac{{\left( {x_{1}^{2} + y_{1}^{2} } \right)\left( {x_{3} - x_{2} } \right) + \left( {x_{2}^{2} + y_{2}^{2} } \right)\left( {x_{1} - x_{3} } \right) + \left( {x_{3}^{2} + y_{3}^{2} } \right)\left( {x_{2} - x_{1} } \right)}}{{2\left( {x_{1} \left( {y_{2} - y_{3} } \right) - y_{1} \left( {x_{2} - x_{3} } \right) + x_{2} y_{3} - x_{3} y_{2} } \right)}},$$

6 $$r = \sqrt {(x_{1} - x_{0} )^{2} + (y_{1} - y_{0} )^{2} } .$$

All three quantities are functions of $x_{3}$.

In addition, the angle $\theta$ between the points $P_{1} ,P_{2}$ and the centre can be found by the dot product of the vectors $\left( {x_{1} - x_{0} , y_{1} - y_{0} } \right)$ and $\left( {x_{2} - x_{0} , y_{2} - y_{0} } \right)$, i.e.,:

7 $$\theta = \text{Arccos} \left( {\frac{{\left( {x_{1} - x_{0} } \right)\left( {x_{2} - x_{0} } \right) + \left( {y_{1} - y_{0} } \right)\left( {y_{2} - y_{0} } \right)}}{{\sqrt {(x_{1} - x_{0} )^{2} + (y_{1} - y_{0} )^{2} } \sqrt {(x_{2} - x_{0} )^{2} + (y_{2} - y_{0} )^{2} } }}} \right).$$

As a result, the length $L$ of the curve section will be:

8 $$L = \theta r,$$

and through the use of (4)–(7) will be a function of $x_{3}$. At the same time, the slack, $L_{\text{s}}$ can be calculated as:

9 $$L_{\text{s}} = L - \left( {y_{1} - y_{2} } \right)$$

hence the slack can be expressed as a function of $x_{3}$. This allowed the fabric excess measurement ($L_{\text{s}}$) to control the design of the graft (with the appropriate definition of $x_{3}$).

### Endograft Model

The initial configuration of the endograft is dictated by the position of the iliac legs relative to the body (how low and under which angle does the locking happen). Given these parameters, in our framework, the rings and fabric of all modules are built in the desired orientation, before the initiation of the analysis.

**Figure 4:**
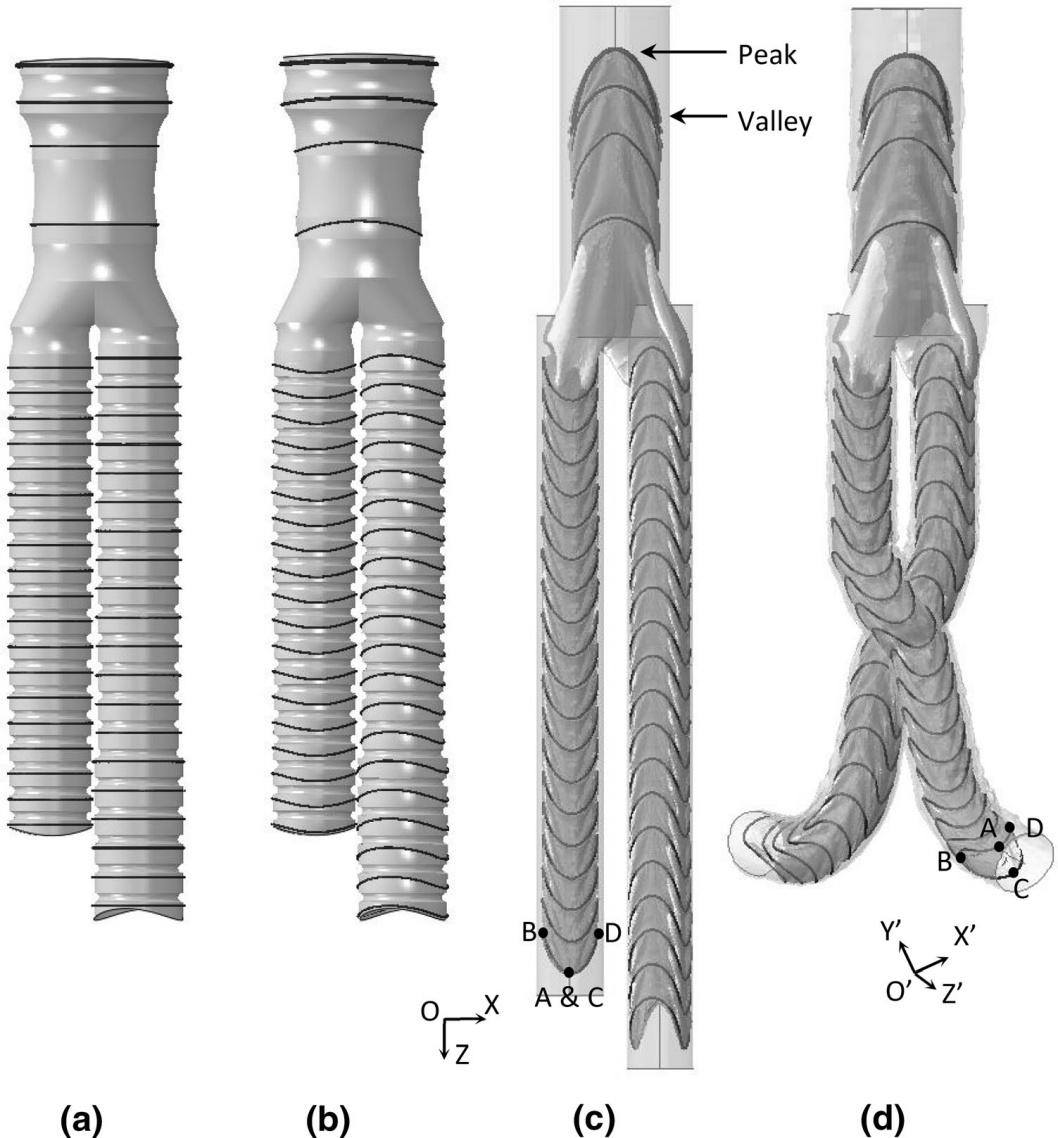
The Anaconda™ model at the beginning of the analysis (a) and after fabric initialization (b). Pulling forces fit each module inside its corresponding pair of catheters (c) which are then moved during delivery according to the vessel’s centre points (d). The saddle points and coordinate systems of a representative ring stent bundle are illustrated. Points A, B, C, D and points A′, B′, C′, D′ are overlapping.

The connection between the fabric and the rings occurs in the first two steps of the analysis. In the initial step, a sinusoidal displacement is assigned to the rings to acquire the saddle shape the rings have at their rest state (Fig. 4b). At this stage, frictionless contact is used with a separation restriction in the normal direction, to establish the fabric-ring contact. After that, the rings are partially compacted by being further pulled (Fig. 4c) and the contact is changed to “rough” (a Coulomb frictional model with infinite friction), in order to secure fixation. This condition remains for the rest of the analysis. Note that the first step of the process is, in essence, part of the second step (i.e., the fabric-ring contact occurs during ring compaction), hence the computational cost of it does not burden the total runtime.

### Catheters

For the delivery of the device into the abdominal aorta, catheters are used and require to be modelled to simulate correct stent positioning. More specifically, for each endograft module, a pair of cylindrical catheters (shell elements, S4R) is constructed, one with a rigid wall (Catheter A) and one inflatable (Catheter B). The two catheters of every pair have the same initial radius and are tied to each other at the beginning of the analysis. Catheter A is also tied to a centreline (made of linear beam elements, B31) that controls delivery, while Catheter B has no centreline and aids deployment.

The rings are pulled at the saddle points, enough to fit inside the catheters, and are slightly pulled apart from each other, with a displacement $L_{\text{compact}}$. This displacement correlates to the elongation of the device when placed inside the catheter, during the manufacturing process.

Once the rings are inside the catheters, the pulling forces are supressed, hence the rings are released and allowed to come into contact with Catheter A. Delivery follows, as boundary conditions are applied to the centreline of Catheter A, forcing both catheters to move towards the centreline of the target vessel (Fig. 4d). Once delivery is completed, the endograft switches its contact from Catheter A to B, the tied contact between the catheters is supressed and pressure is applied to the inner wall of Catheter B making it expand, thus allowing the endograft to deploy inside the vessel.

**Figure 5:**
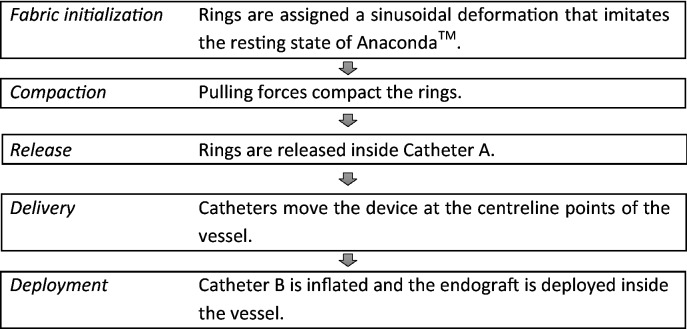
The steps of the EVAR simulation.

The overview of the analysis is described in Fig. 5.

### Boundary Conditions

As can be seen in Fig. 4, the original and the final orientation of the rings do not coincide. Furthermore, the final orientation of each ring is unknown prior to the import of the vascular geometry. This poses a challenge in the definition of boundary conditions at the final stage of the simulation, since (in Abaqus) no node can have boundary conditions expressed in more than one coordinate system, even if they are applied in different steps of the simulation.

To address the issue, reference points were created at and tied to the peaks and valleys of each ring. This way, the reference points and the ring’s peaks/valleys occupied the same space and shared all translational and rotational degrees of freedom from the beginning of the analysis. During compaction, boundary conditions were applied at the peaks and valleys of each ring (points A, B, C, D) making use of local coordinate systems oriented according to the original endograft positioning. However, after delivery, boundary conditions were applied at the reference points of each ring (points A′, B′, C′, D′) making use of individual coordinate systems oriented according to the centreline of the vessel.

*Via* these manipulations, the following equations were defined (refer to Fig. 4 for point definitions):

10 $$\begin{aligned} {\text{Rotation}}_{\text{X}} = 0{\text{ for points B}},{\text{D}} \hfill \\ {\text{Rotation}}_{\text{Y}} = 0{\text{ for points A}},{\text{ C}} \hfill \\ {\text{Rotation}}_{\text{Z}} = 0{\text{ for points A}},{\text{ B}},{\text{ C}},{\text{ D}} \hfill \\ {\text{Displacement}}_{\text{X}} = 0{\text{ for points A}},{\text{ C}} \hfill \\ {\text{Displacement}}_{\text{Y}} = 0{\text{ for points B}},{\text{ D}} \hfill \\ {\text{Displacement}}_{\text{Z}} = 0{\text{ for points B}},{\text{ D of the most proximal ring of each module}} \hfill \\ \end{aligned}$$

at coordinate system O during compaction and O′ during release and deployment. Note that during delivery, the device was constrained only by the catheters and not implicitly by boundary conditions.

From deployment onwards, Rotation_Y_ = 0 was also applied to points B, D; these boundary conditions increased the stability of the analysis, without altering the shape of the deployed rings. Appropriate boundary conditions were also applied to the catheters throughout the analysis to prevent rigid body motion.

Various contact conditions were used throughout the analysis. Apart from the fabric/ring and catheter/catheter contact, self-contact was assigned to the fabric (with a friction coefficient of 0.005) and the rings (frictionless). In addition, appropriate contact was assigned to various steps of the analysis to release the rings inside the catheters and perform delivery. At deployment, contact was assigned between the vessel and both the rings (friction coefficient of 0.5) and the fabric (friction coefficient of 0.005). The friction coefficient values were chosen during the conduction of preliminary studies.

### 3D Printed AAA Validation

To assess the performance of the model, validation was conducted in a 3D printed AAA. Experimental and virtual deployment of an Anaconda™ took place and the spatial difference between the two approaches was used as an error indicator for the model. The key outputs of interest were the agreement at the proximal and distal regions of the endograft, the macroscopic shape of the flow lumen and the existence of sharp fabric folds, as these dictate the global and local shape of the fabric, and therefore are of greatest interest to the resulting CFD analysis.

For the construction of the AAA, a CAD model of a healthy aorta was manipulated appropriately, an aneurysm was added and 3D printing was performed using the transparent resin WaterShed^®^ XC 11122. The AAA model was cable-tied to a peg board and the deployment was conducted *via* catheters, following the steps indicated in the device’s Instructions for Use. The specifications of the Anaconda™ modules (illustrated in Fig. 2d) are reported in Table 2. Finally, the sharp ends of the hooks were removed from the endograft as they could not penetrate the resin and would cause the rings to be deformed unnaturally.

**Table 2:** The Anaconda™ module specifications used in the 3D printed AAA validation.

Module name	Number of rings	Ring diameters [mm]	Wire diameters [mm]
Body (OLB23)	4	22.43, 22.24, 18.59, 18.59	0.18, 0.18, 0.16, 0.16
Iliac leg (L12x100)	22	12.25 for all	0.16 for all
Iliac leg (FL1213x110)	16 + 6	12.25 and 13.31	0.16 for all

This configuration was replicated in the numerical environment and the aorta was modelled as rigid. Thanks to a semi-automated python script, the set-up of the FEA model was achieved in approximately 1 h and the total number of elements used was 235,000. The analysis run on 12 cores (Intel^®^ Xeon^®^, 3.4 GHz) with 64 GB RAM while the ratio of kinetic over internal energy was monitored to be kept below 10% (at the end of the simulation was 2.4%), ensuring the negligibility of inertia.

Finally, the comparison of the experimental and FEA results was performed by superimposing images of the two in four different views (front, back, left and right).

### CFD

The ultimate objective of the study was to utilize the FEA simulation for a hemodynamic analysis, able to capture the effect graft wrinkles have on the flow field. To achieve that in the simulation, the deployed endograft of the previous section was pressurized at a mean arterial pressure $P_{\text{m}} = 12.44$ kPa (93.3 mmHg). Note that the rigid walls of the AAA model resulted in a most-wrinkled fabric geometry, despite this pressurisation. The graft fabric was then exported from Abaqus in .STL format and introduced to a series of software for necessary treatment.

More specifically, the exported geometry was introduced to the open-source mesh processing software MeshLab for mesh simplification and smoothing. Subsequently, the geometry was introduced into Materialise 3matic (Materialise NV, Leuven, Belgium) where an inwards offset, representing the fabric thickness, was applied to the fabric to resolve small self-penetrations and to isolate each iliac leg. Moreover, the inlet and outlets were extended (4 times the inlet radius and 5 times the outlet radii, respectively) using straight tubes to enable the application of a parabolic velocity profile at the inlet, and to enable the flow to develop in the regions of interest. Finally, the processed fabric geometry was re-introduced into Abaqus for the CFD analysis (Fig. S2 of Electronic Supplementary Material).

Abaqus/CFD solver (version 6.13-2) is a commercially available computational fluid dynamics package which has been previously used in the modelling of the haemodynamics of the aorta.^32^ The solver uses the integral form of the conservation equations, with the solution of the incompressible Navier–Stokes equations achieved using a second order accurate semi-implicit projection method and a linearly complete second-order accurate least-squares gradient estimation method. A node-centred finite-element discretization was used for the pressure and a cell-centre finite volume discretisation of all other transported variables (such as velocity) was adopted. A second-order accurate time integration was used, with all diffusive terms, advective terms and body forces integrated *via* the Crank-Nicolson method. Automatic time incrementation was used, with the increment size continually adjusted to satisfy the Courant-Friedrichs-Lewy stability condition (CFL = 0.45) for advection. Spatial discretisation was achieved using the default free meshing algorithm with tetrahedral elements. The blood flow was assumed to be laminar, in keeping with other studies in the field.^6^,^12^,^37^ Additionally, the flow was assumed to be incompressible and the blood non-Newtonian with density $\rho$ = 1060 kg/m^8^ and dynamic viscosity described by the Carreau-Yasuda model:

11 $$\mu = \mu_{\infty } + \left( {\mu_{0} - \mu_{\infty } } \right)[1 + (\lambda \dot{\gamma })^{0.64} ]^{{\frac{n - 1}{2}}} ,$$

where $\dot{\gamma }$ denotes the scalar shear rate. The shear viscosity at low shear rates, $\mu_{0}$ = 0.16, the shear viscosity at large shear rates,$\mu_{\infty }$ = 0.0035 and material coefficients $\lambda$ and $n$ were equal to 8.2 s and 0.2128 respectively, following common values found in the literature.^20^,^34^

Three cardiac cycles were simulated for pulse cycle independency, with a parabolic velocity inlet. More specifically, velocity was defined along the longitudinal axis *z*, as:

12 $$v_{z} \left( {t,r} \right) = v_{ \hbox{max} } \left( t \right)\cdot f\left( r \right),$$

where $v_{ \hbox{max} } \left( t \right)$ was measured in m/s and followed the pulse as described in Fig. S3 (Electronic Supplementary Material) providing a mean flowrate of 2.5 L/min for 60 bpm, while the profile $f$ was defined as:

13 $$f\left( r \right) = 2\left( {1 - \frac{{r^{2} }}{{r_{0}^{2} }}} \right),$$

with $r_{0}$ being the radius of the inlet. The pressure outl*et al*so followed a natural waveform (Fig. S3) and varied between 83 and 122 mmHg. Note that these conditions represent only one scenario of the possible boundary conditions present in the human aorta. Additionally, a no slip condition was applied at the walls. A mesh convergence study of a uniform mesh was performed, ensuring that both the velocity and the time averaged wall shear stress (TAWSS) did not change more than 3%. 7 million 4-node linear tetrahedron (FC3D4) elements with an approximate size of 0.25 mm were used for the simulation and the analysis run on the same computing set-up as before.

Results for the velocity field, the vorticity and the TAWSS were computed because of their medical relevance; particularly the last two variables have been linked to ILT formation,^2^,^39^ an unwanted phenomenon, especially when developed in the narrow regions of the endograft (i.e., the iliac legs). Note that TAWSS is defined as the integral of the magnitude of wall shear stress, $\overrightarrow {{\tau_{W} }}$, over the third cardiac cycle $T$:

14 $${\text{TAWSS}} = \frac{1}{T}\mathop \smallint \limits_{0}^{T} \left| {\overrightarrow {{\tau_{W} }} } \right|dt.$$

Moreover, the oscillatory shear index (OSI) and the Relative Residence Time (RRT),^6^ which are derived from the wall shear stresses, were used as further indicators of the increased risk of thrombus formation. For the rest of the variables, output was retrieved at the maximum or minimum inlet velocity of the 3^rd^ cardiac cycle. The set-up time of the CFD analysis was approximately 1.5 h.

## Results

### Fabric Testing

The average distance between R1–R2 and R3–R4 at the full extension state was calculated to be 15.2% higher than the rest state. This value was used as a percentage of the slack approximation $L_{\text{s}}$ for all curved fabric sections.

### 3D printed AAA validation

The 3D printed vessel was a very stiff structure, adding complexity to the delivery of the device. This rigidity, in combination with the sharp (90°) turn at the iliacs proved challenging for the delivery system and an unusually high force was needed to introduce the catheter. All three delivery systems (for the three modules) were twisted and pushed to make the initial turn, and the body section experienced minor “jumps” at the time of deployment. The result of this strenuous delivery was the twist of the device, so the left leg had to be connected to the right iliac while the right leg to the left one. Nevertheless, such an endograft position can also occur in clinical practice if the iliacs are too tortuous. Because of that, the validation was pursued with this set-up, which can be thought of as a model of a challenging EVAR deployment.

**Figure 6:**
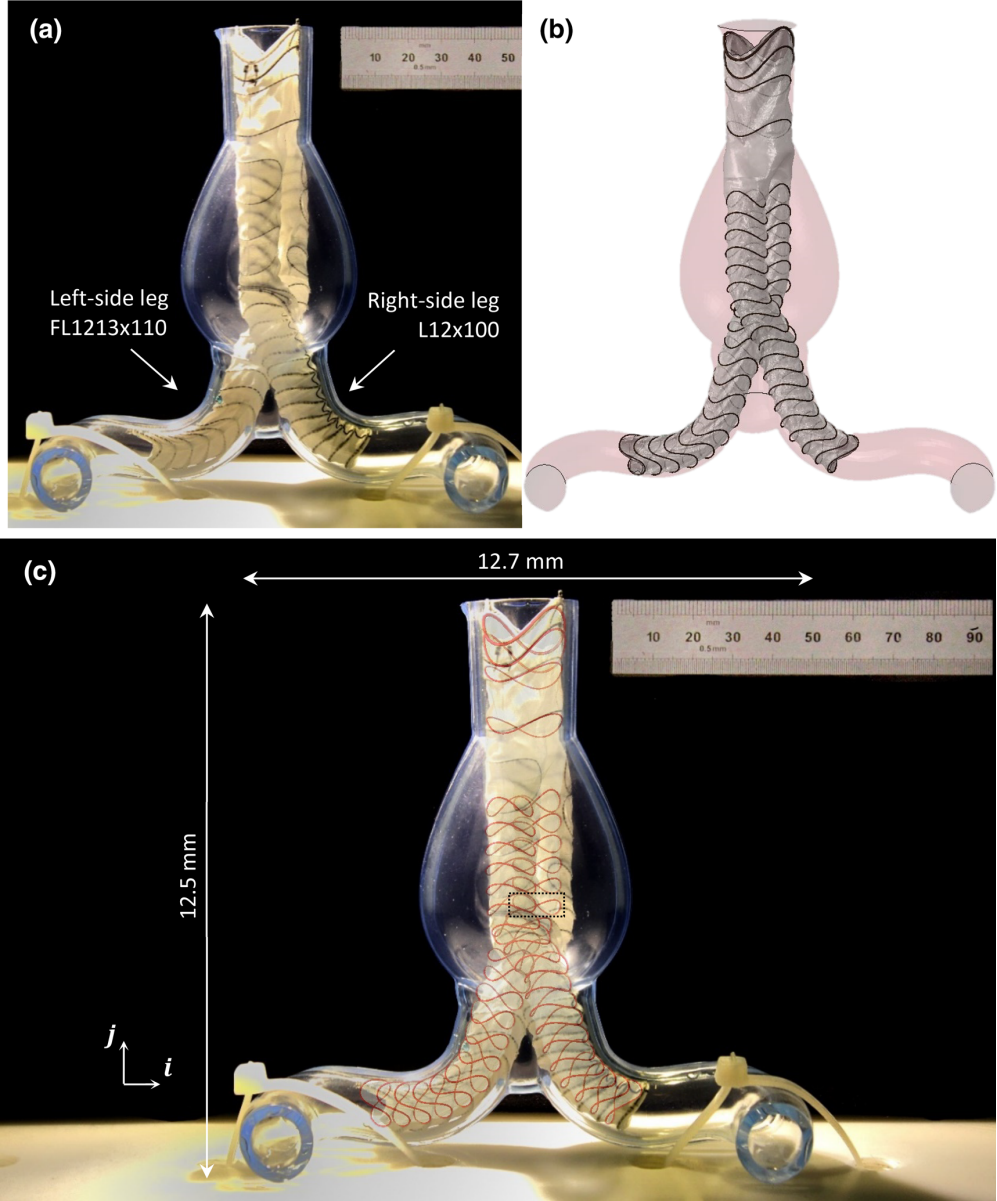
The Anaconda™ device experimentally (a) and virtually (b) deployed inside the mock artery. At the superposition (c), the FEA rings are highlighted in red.

The maximum error for the FEA model, defined as the maximum 3D distance between the experimentally and the virtually deployed rings, was calculated by superimposing images from the front, back and lateral sides (for the front side, see Fig. 6). Since the pictures were taken from 2 perpendicular planes, the 3D distance between any two locations can be expressed as $L = \sqrt {a^{2} + b^{2} + c^{2} }$, with $a$, $b$, $c$ being the components of this distance in the $i$, $j$ and $k$ directions.

Analysis on the areas with the greatest discrepancy showed that the maximum error was located at the anterior peak of the sixth ring of the left iliac leg (the black box in Fig. 6c). The 3D distance between the FEA peak and the experimental one was identified to be 12.6 mm.

It was generally observed that the aneurysmal region was the area of greatest errors. This comes as no surprise, since in this area, stent rings are unsupported. In reality, during delivery, endografts follow the path of least resistance and not the one described by the centrelines of the vessel. This has obvious implications in the deployed position of the device, and in more tortuous aneurysms will generate even higher errors. Yet because in the aneurysmal region endografts are less restricted, they experience low angulations, hence the accurate prediction of this section is less important from a structural perspective.

The discrepancies produced at the high contact regions were much lower. The last ring of the right-side iliac leg was chosen as a representative case of a high-error ring in the non-aneurysmal site and the 3D distance between the FEA peaks and the experimental ones was measured to be 3.4 mm. This value is below the 5 mm threshold that is used as a target for the accuracy. It is interesting to note that part of this error corresponds to the inability of the model to capture the rotation along the longitudinal direction of each module. For example, the FL1213x110 leg twists along its length as can be seen in Fig. 6c. This is most probably the result of unsheathing and compaction, and is not replicated by the FEA model.

**Figure 7:**
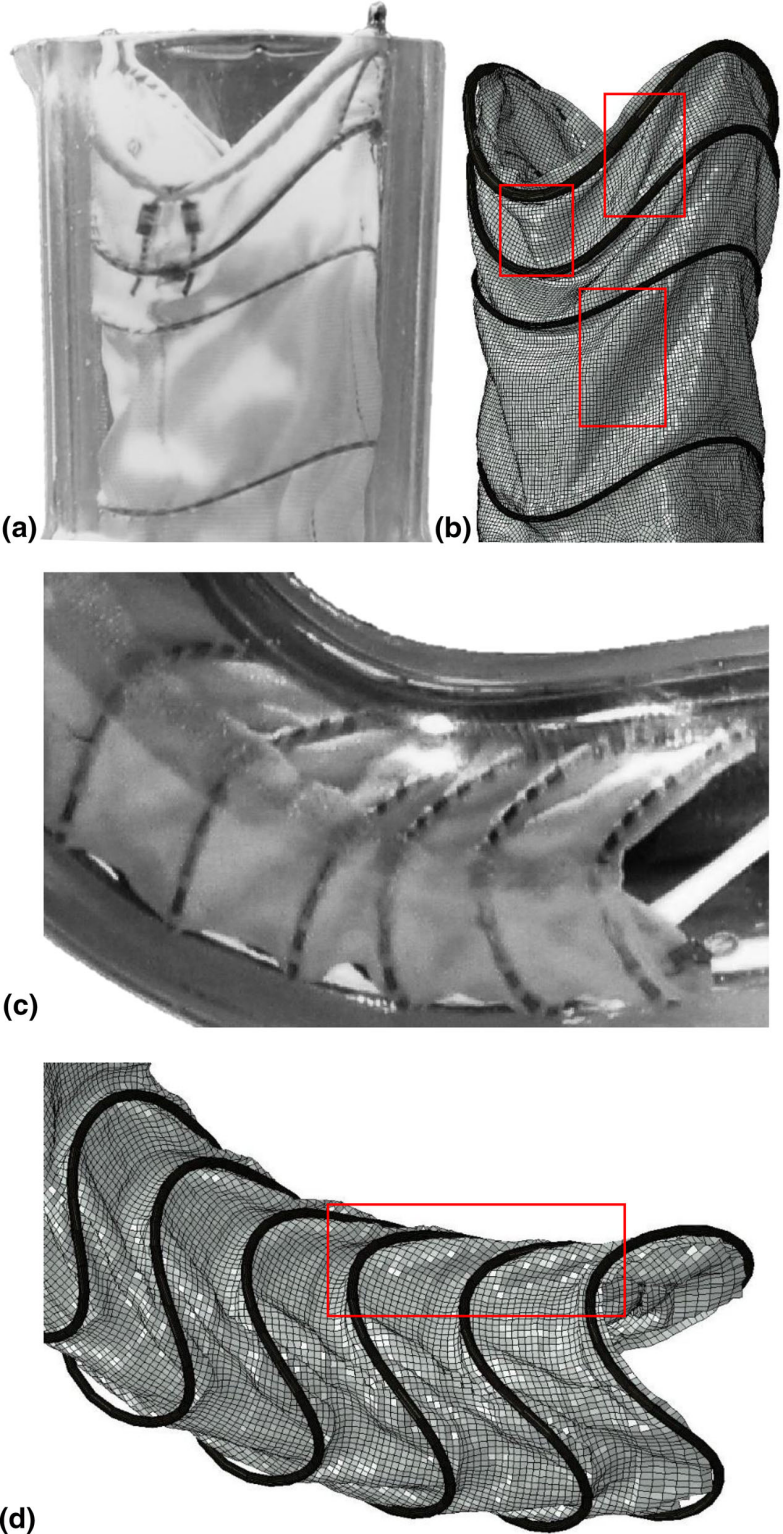
Detail of the proximal (a & b) and distal (c & d) regions of the endograft. The fabric folds are visible both for the body and the left-side iliac leg modules while the highlighted regions are well predicted regions of high wrinkling. In (c) & (d) the axial twist mismatch is also visible.

Regarding the behaviour of the fabric, the folds evident in the deployed device have been reproduced. The accuracy of them is not quantified but it can be seen that, in most cases, folds tend to occur along common lines for both the experiment and the simulation. Furthermore, intense wrinkles that can affect flow have been correctly represented, as illustrated in the highlighted areas of Fig. 7.

In general, the simulated locations of greatest interest (proximal and distal regions of the endograft) demonstrate good agreement with the experimental regions, while the macroscopic shape of the flow lumen has been accurately captured for the entire length of the device.

Finally, the analysis time was 4 h 14 m, a result much faster than the times reported in the literature (> 40 h). A video of the structural analysis is presented in the Electronic Supplementary Material (S4).

### CFD

**Figure 8:**
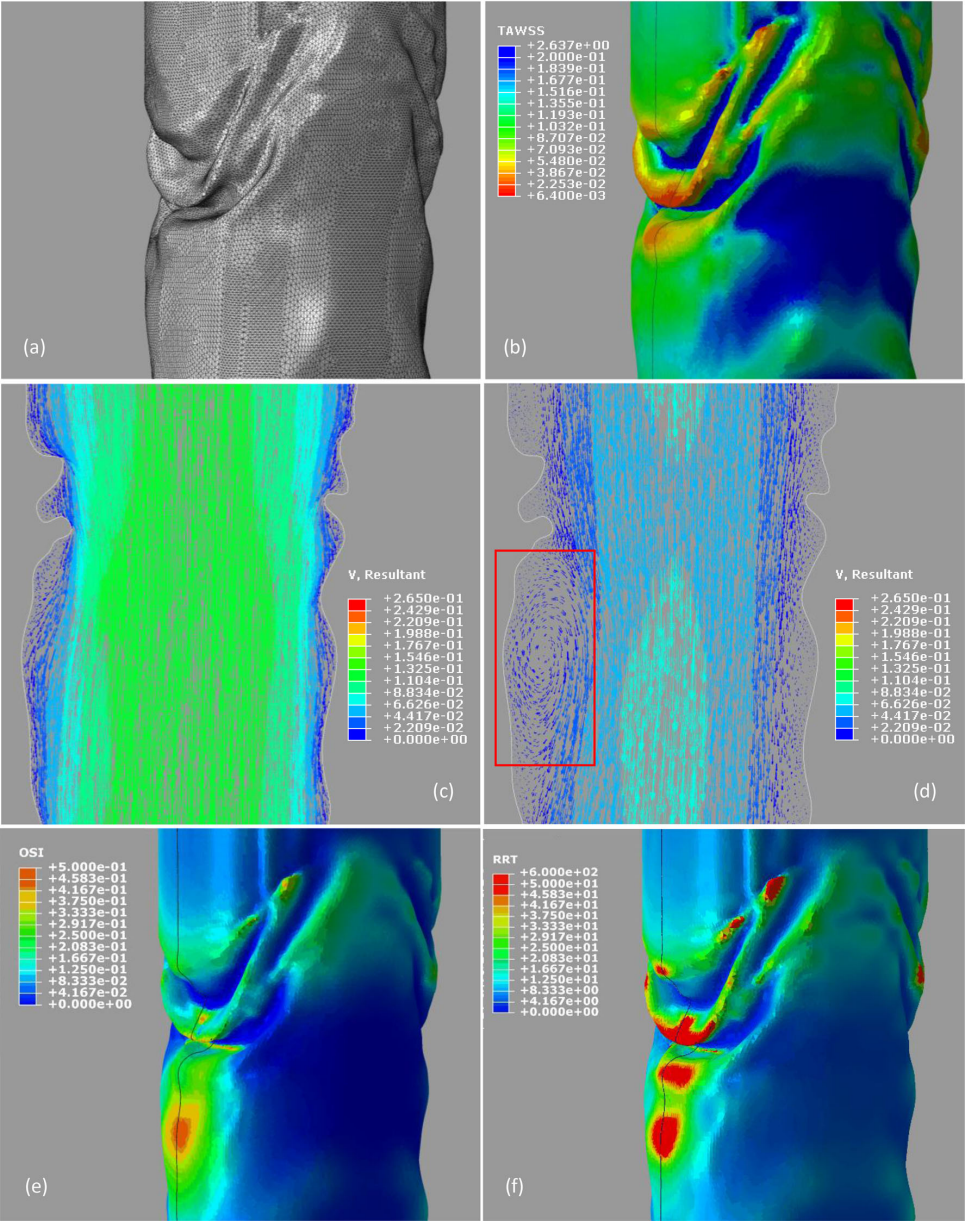
Detail of the fabric folding between the first and third proximal rings of the endograft. The mesh (a) and the TAWSS (Pa) (b) are illustrated. The cut-through line visualized in (b) highlights the cut plane used in the images (c) and (d), where the velocity vectors (m/s) during the maximum velocity inlet and minimum velocity inlet are presented respectively—note that recirculation can be observed at the left part of image d (red box). At the bottom, the OSI (e) and the RRT (f) are illustrated, during the 3^rd^ cardiac cycle as well.

The velocity produced a smooth output macroscopically. This is in keeping with studies of the haemodynamics of the Anaconda graft reported in literature which did not account for the effect of fabric folds.^12^ However, when closely examining the folds, the effect of the wrinkles on the blood flow becomes evident. In regions with large folds, the velocity of the blood decreases dramatically leading to areas of almost stagnant flow, even when the inlet flowrate maximizes (Fig. 8c). Moreover, when the velocity of the inlet reaches its minimum, flow recirculation occurs (Fig. 8d). A similar behaviour can be observed in smaller fabric folds too, yet the effect is less strong.

In order to determine the impact that the recirculation zones and stagnant flow has on the risk of thrombus formation, the TAWSS was also studied and compared against the values reported in the literature. Magnetic resonance velocity mapping in the infrarenal aorta found the mean wall shear stress in six healthy volunteers to be 0.28 ± 0.01 Pa,^23^ while 4D flow measurement of the thoracic aorta of 224 patients found TAWSS values of 0.87 ± 0.31 Pa and 0.90 ± 0.37 Pa in the ascending and descending aorta respectively.^4^ When considering the effect of TAWSS on the risk of thrombus formation, Myerson 18 proposed that a wall shear stress of < 0.2 Pa stimulated smooth muscle cell proliferation in venous bypass grafts and was therefore a critical threshold for the occurrence of neointimal thickening. Therefore, in this study a threshold value of 0.2 Pa for TAWSS is adopted when considering the increased risk of thrombus formation.

**Figure 9:**
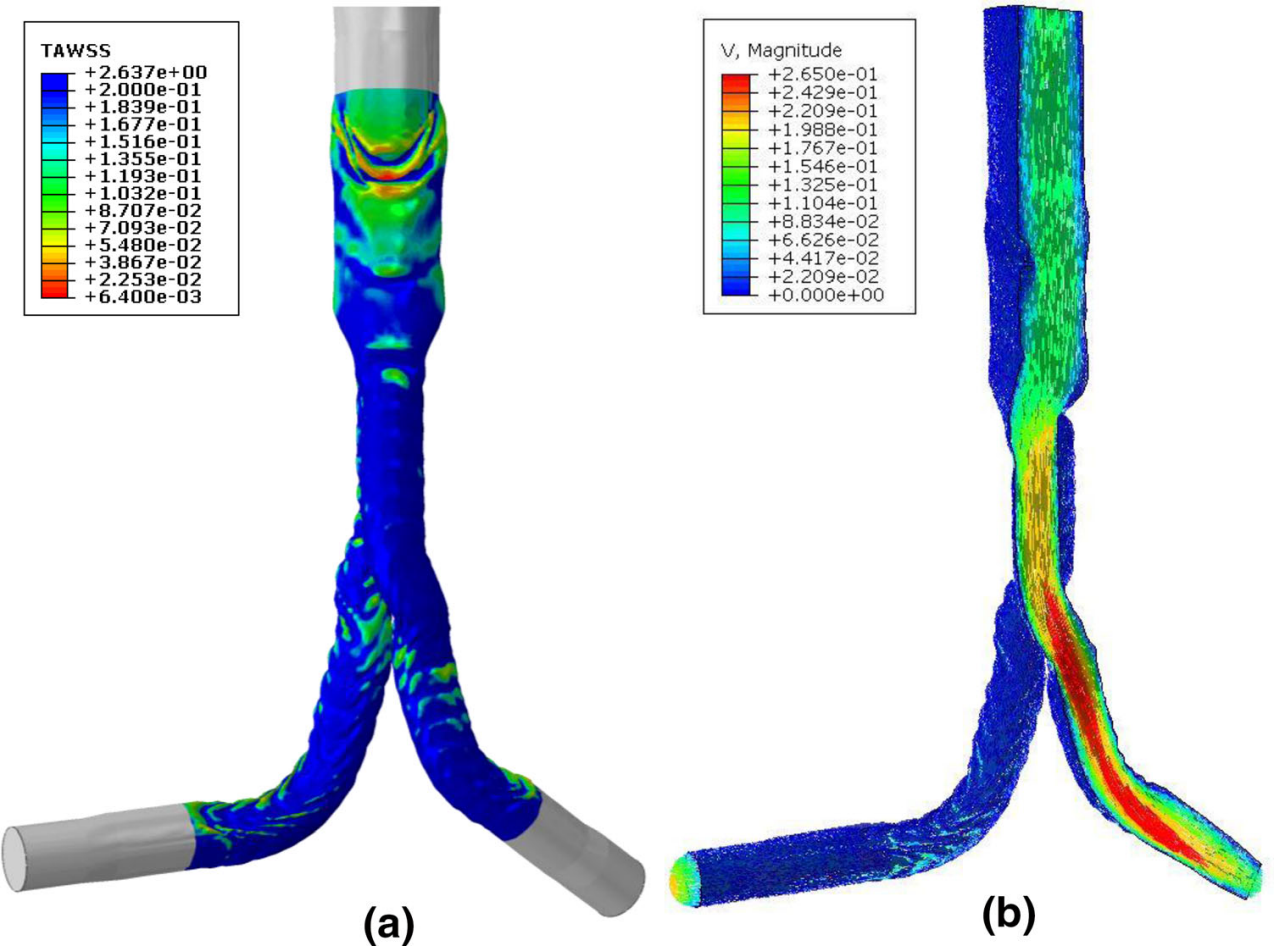
The TAWSS (Pa) (a) and the velocity vectors (m/s) and contour plots, on a cut plane of the model, at the time of maximum inlet velocity (b).

Considering the TAWSS plots shown in Figs. 8b and 9a, the impact of the folds becomes evident. The large regions of fabric which fold inwards towards the lumen of the graft create a drop in flow velocity in the regions immediately above and below the infold, resulting in a drop in TAWSS to near zero in these regions and increasing the risk of thrombus formation (Fig. 8b). As a result of the reduced TAWSS and oscillating flow, these regions also show high OSI (maximum of 0.46) and RRT (> 50 Pa^−1^ in larger folds, rising locally to as high as 600 Pa^−1^) (Figs. 8e and 8f, respectively). While these values of peak OSI and RRT are significantly higher than those previously reported in literature,^6^ it must be noted that these studies did not include the effects of graft fabric and stent folding. In contrast, regions with no folds and those with inwards folds experience physiological TAWSS stress values of > 0.2 Pa, OSI < 0.1 and RRT < 10 Pa^−1^, indicating that these regions are at a reduced risk of thrombus formation. The vorticity contour plot was qualitatively similar to the TAWSS plot, and therefore is not presented in this work.

The CFD analysis runtime was 12 h.

## Discussion

In the current work, a computationally efficient Anaconda™ model was developed, able to closely recreate the post-deployment position of the stent-graft. The calculation of the overall shape of the device, given the position of each module in its proximal landing zone, can eventually lead to successful EVAR predictive tools and someday aid surgical planning. At the same time, it can enhance the design of patient specific stent-grafts, as their use has been demonstrated to result in better vascular deployments,^29^ hence more promising outcomes. Most importantly, though, the developed model was shown to create high-fidelity FEA results which can provide detailed lumen geometry for CFD analysis. Such methodology can be used both as a design tool (by simulating different proposed endograft designs in the same anatomy) and as a clinical tool, to highlight hemodynamic risks of already treated patients. Post-op analyses could be further improved by directly matching the FEA stent rings’ positions to follow-up CT scans, capturing the fluid domain (i.e., fabric layout) in a detail which is far beyond conventional CT scans.

In our framework, the Nitinol rings of the device were modelled following an ‘equivalent beam’ approach and a simple elastic material was used. Nevertheless, since the analysis was displacement driven for the greatest part of the EVAR simulation and the anatomy model was rigid, minimising the impact of the stent’s radial force, such material simplification did not affect significantly the final shape of the rings and the outputs of interest. By altering the $EI$ product (instead of $I$ alone), both thicker wires and a less stiff material were acquired. These effects lead to a coarser mesh and larger stable time increments in the explicit solver respectively, reducing the overall runtime. The computational cost was also reduced by the fabric design, which allowed for a quick ring/graft tie, and the approach of modelling the three endograft modules as one, removing complex module interactions and the necessity for the endograft to be assembled during the analysis.

The model was assessed against an experimental deployment of an Anaconda™ device on a 3D printed AAA and the deployed shape of the numerical and the physical model were compared. For this study, the aortic model used for comparison purposes was chosen to be rigid to minimize the impact of the vessel material model on the analysis. This comparison was not intended as a complete model validation—more cases would be necessary for that—yet its results should be considered indicative of the capabilities of the model.

The FEA and the physical testing images were superimposed, an approach used in the literature for its ease of implementation.^8^,^19^ Measurements illustrated that the prediction of the deployed shape of the endograft in its proximal and distal sections (i.e., the regions of interest) was below the 5 mm error which was used as a reference based on the work of Perrin *et al*.^25^ Part of this error was the result of the inability of the model to take into account the rotation along the longitudinal direction of each module. However, it has recently been shown that rotational effects are able to be captured by FE analyses,^30^ providing encouragement that even greater accuracy could be achieved in the future. Nevertheless, since the act of stent deployment (even in lab conditions) is not highly repeatable, all models will unavoidably generate some errors when compared to post-op images. Taking into account that even in simple bending tests, the positional error of stents can reach up to 4.0 mm,^10^ one can better appreciate the technical difficulties involved in creating an accurate FEA representation of EVAR.

The most critical aspect of the structural model is its cost effectiveness. The full creation, compaction, delivery and deployment of the endograft in the vascular section yielded results in just over 4 h, reducing the runtimes achieved in the FEA literature of EVAR by an order of magnitude.

The reliable global shape, along with the efficiency of the model, allowed the ultimate goal of the study to be pursued: a detailed hemodynamic analysis. Unfortunately, buckling and kinking incidents are not rare for endograft devices,^5^ especially in tortuous arteries.^7^ Such events can result in flow disturbances, thrombosis and eventually to ischemia,^17^ jeopardizing the health and life of EVAR patients. As a result, the in-depth understanding of the consequences the deployed endograft shape has to the hemodynamic is crucial, yet not trivial to acquire.

In the vast majority of CFD studies, the EVAR devices are simulated with an unwrinkled fabric surface that serves as a smooth boundary wall for the blood flow.^20^,^22^,^34^,^35^ Yet by disregarding the wrinkles on the surface of the graft, possible flow disturbances can be overlooked. Herein, it was successfully demonstrated that the output of the FEA simulation can be used as input for a CFD investigation. Although the fabric model wasn’t optimized for a hemodynamic analysis, it was demonstrated that a high level of detail can still be achieved. In this study, the overall flow appeared smooth, perhaps because endograft devices with crossed legs can promote a favourable hemodynamic effect.^33^,^34^ The peak velocity occurred at the iliac legs, as reported in previous studies as well.^28^ Close to the boundary though, the existence of fabric folds was shown to affect the hemodynamic fields of the stented AAA. Major wrinkles disturbed the flow pattern and caused recirculation and low wall shear stresses, effects that have been linked to thrombus formation.^26^ It is interesting to note that after EVAR, endovascular devices continue to expand within the native artery to near nominal oversize,^14^ meaning that the rigid geometry used in this study represents a worst case for fabric infolding. In the future, this CFD analysis capability will be validated against experimental data to strengthen its use as a tool in designing new stent grafts to promote smoother flow. Furthermore, an inhomogeneous mesh (coarser mesh in the centre of the lumen) should be promoted to improve the computational performance, yielding results even faster.

Given the complexity of the simulation, a series of assumptions were necessary to be implemented in the study. Firstly, the use of a rigid arterial wall is a limitation and although the impact of wall elasticity has been shown to be minimal in the global positioning of the deployed device,^25^ local implications are visible (see for example the end rings of the right leg in Fig. 6 that do not exhibit as intense a saddle deformation as the ones in the simulation); as Derycke *et al*.^11^ noticed, under a rigid deployment, stent rings can be radially and longitudinally restricted due to strict geometric constraints. Nevertheless, it was deemed that a non-rigid wall would add significant complexity to the model, making it harder to isolate the amount of error introduced to the virtual EVAR by the endograft model alone. Similarly, the assumption that the centreline of the pre-deployed vessel would be the path followed by the delivery system is an important yet usual simplification (see for example Refs. 1, 9 and 29), that would not hold true when using a realistic vascular material model. Furthermore, the validation of the computational model was based on experimental data of a single, idealized AAA. The use of a realistic, CT based geometry was avoided as the total transparency required for the validation approach chosen could only be achieved post-3D printing, *via* manual treatment, and further geometrical complexity would pose technical challenges. Nevertheless, the use of multiple patient specific case studies will be used in the future for a thorough exploration of the capabilities of the model.

Towards the effort of reducing the computational cost of the FE analysis, the Anaconda™ was treated as a one module device. Despite the fact that the different body-legs position could be taken into account through relative rotations and displacements at the initial stage of the analysis, a direct consequence of the technique is that no overlap could occur between the body and leg sections (i.e., in the docking zone). This limitation can be seen at the bifurcated region of the deployed device (Fig. 6c) where the top rings of the legs have been omitted. Although this approach imposes a limitation to the model, it is believed that the benefit gained from the computational speed far outweighs the minor loss of fidelity in the particular region.

The model presented has been shown to deliver a valuable approximation of the deployed position of the Anaconda™ device in a quick time frame. Furthermore, by implementing the high-fidelity fabric geometry into a blood flow study, the effect of graft wrinkles has been highlighted. The more frequent inclusion of realistic fabric shapes into hemodynamic analyses could bring new insights into the long-term performance of stent-grafts and eventually lead to improved endograft designs and better EVAR practices. Since the smooth-graft approximation cannot incorporate those clinically important characteristics, it is hoped that the current study will bring more attention to the topic and help for more realistic simulations to be conducted in the future.

## Electronic supplementary material

Below is the link to the electronic supplementary material.

Supplementary material 1 (AVI 8546 kb)

Supplementary material 2 (DOCX 1299 kb)
