# Supplementary material for: Efficiently Simulating an Endograft Deployment: A Methodology for Detailed CFD Analyses
Source: Ann Biomed Eng. 2020 May 11;48(10):2449–65. doi: 10.1007/s10439-020-02519-8 (PMC7505889; doi:10.1007/s10439-020-02519-8)

## ELECTRONIC SUPPLEMENTARY MATERIAL


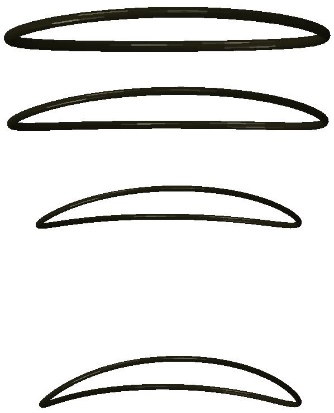


**R1**

**R2**

**R4**

**R3**

Peak

Valley


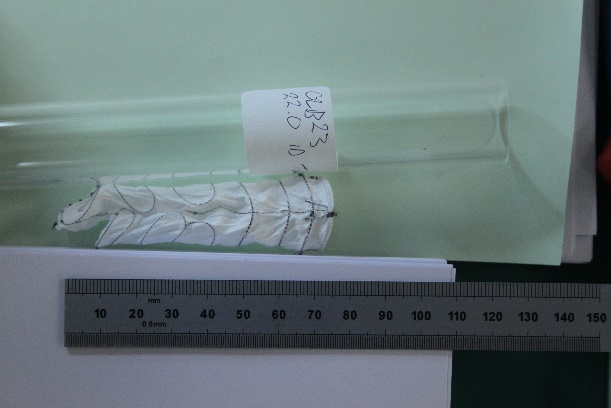


(a) (b) (c)


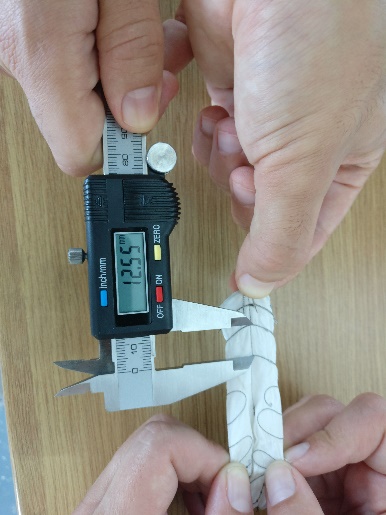


Fig. S1 The distance between R1-R2 and R3-R4 was measured at the peaks and the valleys (jointly named as saddles) of the OLB23 Anaconda^TM^ endograft (a). The resting state (b) and the full extension state (c) were considered.


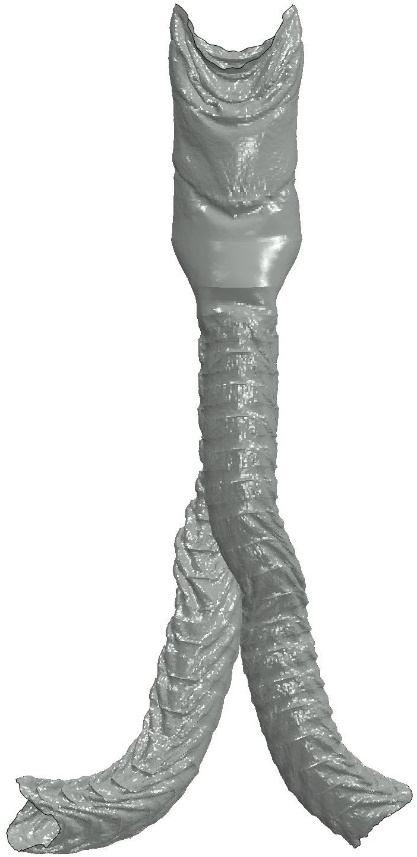

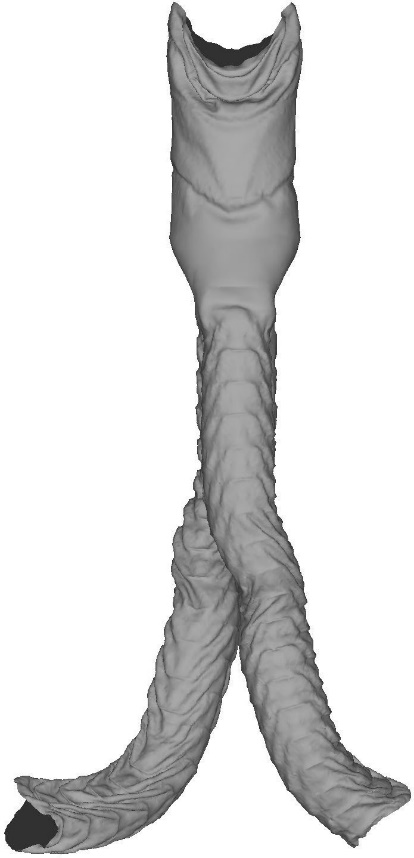

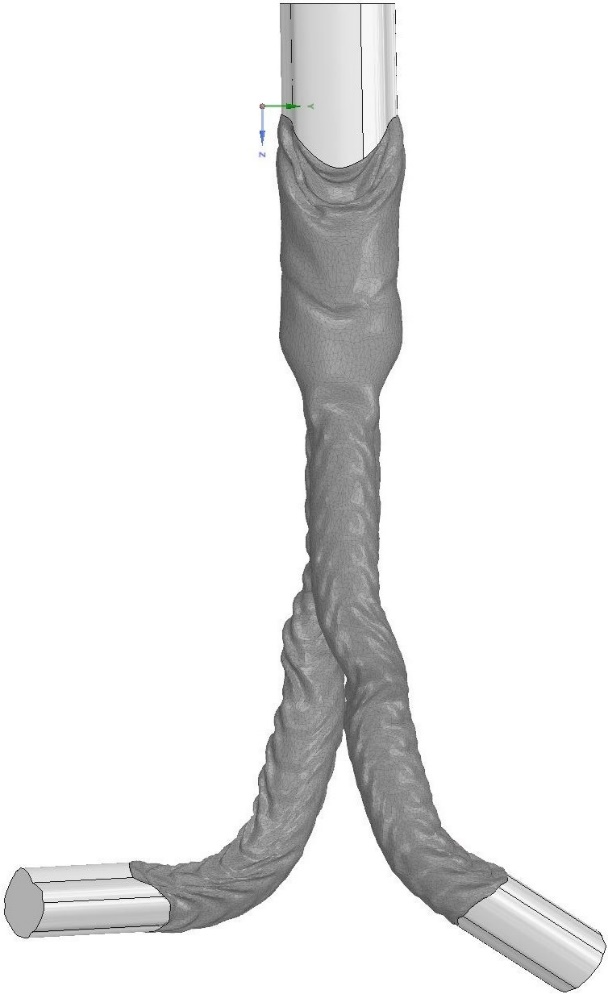

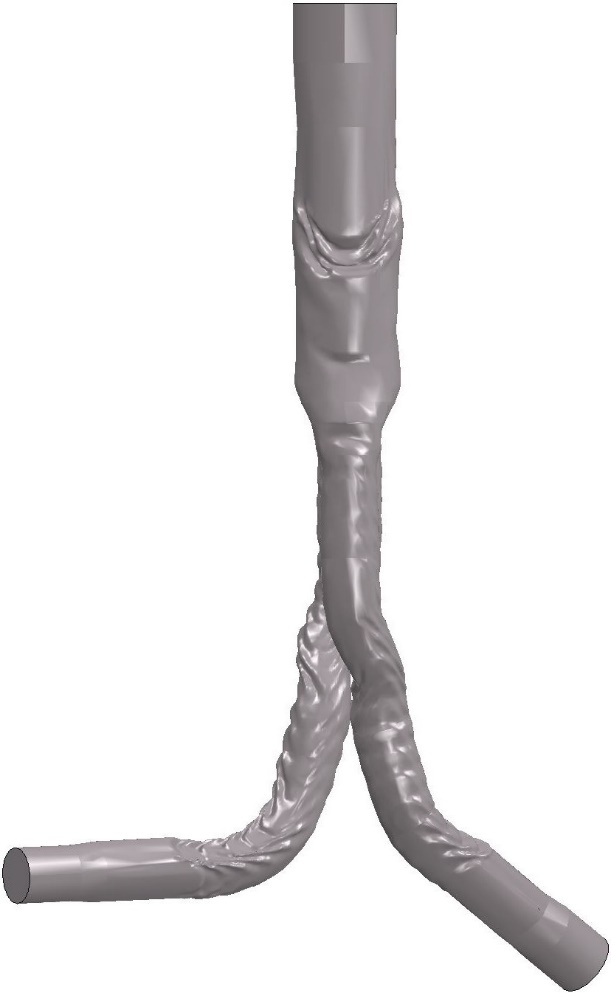


(a) (b)

(c) (d)

Fig. S2 The deformed fabric geometry exported as an FEA result (a), smoothened (b), attached to straight tubes (c) and reintroduced to Abaqus for CFD analysis (d).

Fig. S3 The velocity (left) and pressure (right) waveforms as implemented in the inlet and outlet of the aneurysm respectively. The black marks refer to the time-points of the maximum and minimum velocity inlet of the 3^rd^ cycle, i.e. the time-points of output.


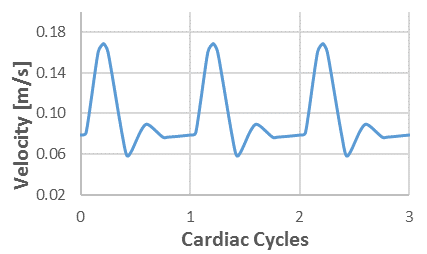

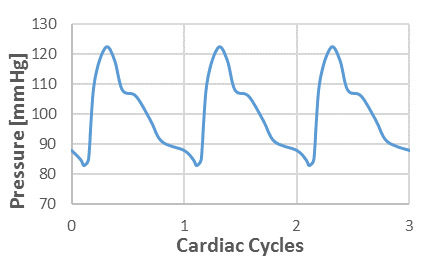

Supplement: Supplementary file 2 — Supplementary material 2 (DOCX 1299 kb) [file 10439_2020_2519_MOESM2_ESM.docx]
